# *Staphylococcus xylosus* Infection in Rainbow Trout (*Oncorhynchus* *mykiss*) As a Primary Pathogenic Cause of Eye Protrusion and Mortality

**DOI:** 10.3390/microorganisms7090330

**Published:** 2019-09-07

**Authors:** Woo Taek Oh, Jin Woo Jun, Sib Sankar Giri, Saekil Yun, Hyoun Joong Kim, Sang Guen Kim, Sang Wha Kim, Se Jin Han, Jun Kwon, Se Chang Park

**Affiliations:** 1 Laboratory of Aquatic Biomedicine, College of Veterinary Medicine and Research Institute for Veterinary Science, Seoul National University, Seoul 08826, Korea; 2 Department of Aquaculture, Korea National College of Agriculture and Fisheries, Jeonju 54874, Korea

**Keywords:** staphylococcal infection, rainbow trout, exophthalmia, immune barrier, pathogenesis

## Abstract

Staphylococcal infections are extensively investigated in humans owing to the resistance of staphylococci to diverse antibiotics commonly used in hospitals. The resistance mechanism of methicillin-resistant *Staphylococcus* *aureus* has garnered the interest of researchers due to its risk to the global public health. Furthermore, the zoonotic potential of staphylococci has led to increased interest in their transmission mechanism via food, livestock, as well as domestic and wild animals. Although fish are globally consumed, there are only few studies on the potential threat of staphylococcal infection in aquatic animals. In this study, we present the first description of *Staphylococcus xylosus* infection and its pathogenicity in rainbow trout, which resulted in fish mortality and economic losses in trout fisheries. We focused on the pathogenic role of the bacterium and its influence on rainbow trout based on the clinical symptoms in the eyes. *Staphylococcus xylosus* infection induced exophthalmia and disrupted the primary immune barrier, which increased the possibility of other secondary bacterial infections in fish under poor conditions, resulting in continuous mortality.

## 1. Introduction

*Staphylococcus* is a Gram positive, spherical, mostly coagulase positive bacterium belonging to the Staphylococcaceae family and order Bacillales [1]. Bacterial cells appear as distinct clusters, like grapes, under a microscope, and hence, the name *Staphylococcus* (“*staphylo*” meaning a bunch of grapes and “*coccus*” meaning spherical formation in Latin) [2]. The genus *Staphylococcus* comprises 11 clusters and includes at least 40 species. They are normally present on the skin and mucous membranes of healthy humans and animals, but their pathogenicity and infection mechanism have gained considerable interest of late, due to their resistance to various antibiotics [3]. Among *Staphylococcus* spp., *Staphylococcus aureus* is extensively studied owing to its methicillin-resistant trait and pathogenicity in humans and animals [3]. In veterinary medicine, *S. aureus* and *Staphylococcus epidermis* are considered the most common primary pathogens causing diseases in various species, including dog, cat, poultry and primates [4,5]. However, information about *Staphylococcus xylosus* is limited, and only a few studies have reported the bacterium as an opportunistic pathogen in animals [6]. *Staphylococcus xylosus* shares its characteristics with other *Staphylococcus* spp., but is a coagulase negative bacterium belonging to the group of *Staphylococcus saprophyticus* [7]. It is also known as a commensal bacterium that generally occurs on the skin and mucous membranes of several animals and considered a nonpathogenic bacteria occurring on rodent skin [8]. Moreover, the bacterium is known to be ubiquitous in nature, persisting in soils and sediments [8]. Although *S. xylosus* is classified as a nonpathogenic bacterium, several studies have reported it as an opportunistic pathogen in animals such as mice and ovine, and its role as a pathogen has been proven using a laboratory mouse model [6]. 

There are a few studies regarding the infection of *Staphylococcus* spp. in fish, but most have focus on the effect of consumption of infected fish in humans and the role of fish as a transmitter of the bacteria to humans [9,10]. Furthermore, there are some reports about *Staphylococcus* infection in aquatic species such as shrimp and sea breams [11], but most studies deal with *S. aureus* and *S. epidermis*, which are also known as major pathogens in humans [9]. Reports on *S. xylosus* as a primary pathogen are scarce in the veterinary medicine field, and none exist in fish. In this study, we report *S. xylosus* as a primary pathogen affecting rainbow trout, causing ocular proptosis, which induces continuous mortality in the fish and leads to economic losses in rainbow trout fisheries.

## 2. Materials and Methods 

### 2.1. Sample Collection

**Figure 1:**
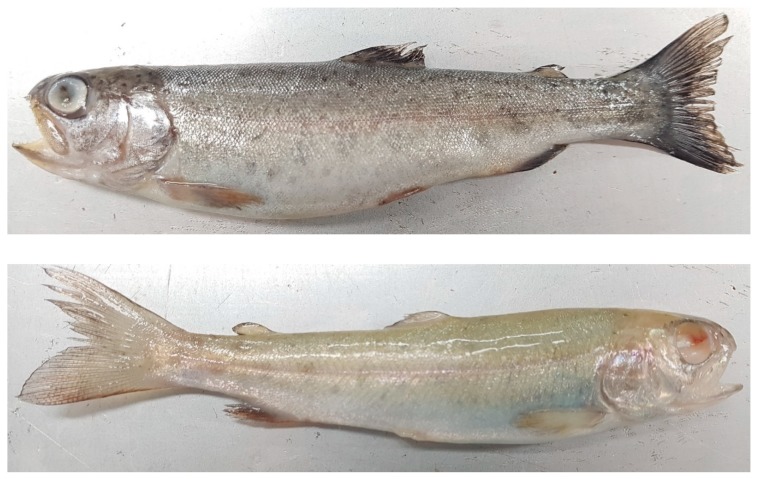
Clinical symptoms of keratitis and exophthalmia observed in *S. xylosus*-infected fish.

In April 2019, one of the rainbow trout fisheries located in Jeonbuk Province, South Korea requested that we diagnose exophthalmia in rainbow trout fries weighing 10 ± 5 g. The farm consisted of six tanks filled with 5.5 tons of water per tank and approximately 2000 fish were maintained in each water tank. The rearing system in the facility comprised a closed circulating water system supplied by ground water, and the water temperature was maintained at 14–15 °C. Diseased fish showed common signs of progressing keratitis and exophthalmia (Figure 1), loss of appetite, and lethargic swimming activity. The mortality rate was around 7% daily, which was relatively low compared to that caused by other bacterial diseases, but the continuous resulting cumulative mortality rate was over 50% at 10 days before antibiotic treatment. Ten fish were sent for examination at our laboratory and all fish were subjected to post-mortem examination.

### 2.2. Examination of Sample and Bacterial Isolation

To rule out infection of any fungi or parasites, the fins and gills of the collected fish were inspected macroscopically. For parasitic examination of the gill, the gill of each fish was smeared on slide glass and examined under a light microscope. Moreover, due to the low water temperature, clinical signs of cold water diseases and bacterial gill diseases were also examined visually [12]. After inspecting external lesions, internal organs including the brain, spleen, kidney, and liver were separated and homogenized in 300 µL of phosphate buffered saline (PBS) for bacterial isolation. As the clinical symptoms mainly occurred in the eyes, the eyeball was separated, and the area connected to optic nerves was smeared for bacterial isolation. There were no signs of any viral infection, but screening for the main viral diseases of rainbow trout such as infectious hematopoietic necrosis (IHN), infectious pancreatic necrosis (IPN) [13], and viral hemorrhagic septicaemia (VHS) was performed by PCR using specific diagnostic primers [14]. For bacterial isolation, 100 µL of the sample homogenized in PBS solution was inoculated on tryptic soy agar (TSA) and brain heart infusion (BHI) agar. The inoculated plates were incubated at 20 °C and 25 °C for 48 h and the dominant colonies were selected for pure culture. Pure-cultured bacteria were maintained in 25% glycerol stock at −80 °C for further investigation.

### 2.3. 16S rRNA Sequencing and Bacterial Identification

To identify pure-cultured bacteria, total genomic DNA was extracted from single colony of the bacteria using the DNeasy^®^ Blood & Tissue Kit (Qiagen, Valencia, CA, USA), following the manufacturer’s protocol. Universal primers targeting the 16S rRNA gene of the bacteria were used for PCR, under conditions reported previously. For gene sequence analysis, the PCR products were sent to the genomic division of Macrogen (Seoul, Korea), where nucleotide sequencing was performed using the ABI PRISM 3730XL Analyzer with the BigDye^®^ Terminator v3.1 Cycle Sequencing Kit (Applied Biosystems, Waltham, MA, USA). The sequence of the 16S rRNA gene was compared to other available 16S rRNA genes by the NCBI blast search and using the EzBioCloud server (https://www.ezbiocloud.net/) to identify similar subspecies of bacteria. For accurate identification of the bacteria, the freshly cultured bacteria on TSA media were used for bacterial identification using MALDI-TOF Biotyper 3 (Bruker, Billerica, MA, USA). In addition, to confirm the identification result, the genomic DNA of bacteria was extracted and analyzed by PCR using specific primers [15] to detect partial *soda*, a house-keeping gene of the bacteria. 

### 2.4. Phylogenetic Analysis

The partial 16s rRNA genes (1458 bp) of 38 closest type strains of *Staphylococcus* spp. were collected to construct phylogenetic trees. Consensus sequences were imported to MEGA 10.0 [16] to analyze and align the sequences using the BioEdit software [17]. The bootstrap analysis was performed across 1000 generations and a phylogenetic tree was constructed using the neighbor-joining method.

### 2.5. Histopathological Examination

To identify histopathological changes caused by the bacterial infection, fish showing clinical signs of corneal opacity were selected and euthanized for examination. As fish with eye proptosis can have a secondary infection, which could affect the histopathological analysis results, they were exempted from diagnosis. The brain of the collected fish was separated, fixed with 10% neutral buffered formaldehyde, dehydrated using ethanol, and embedded in paraffin. The paraffin blocks were sectioned and stained with hematoxylin and eosin, and then the specimens were examined under a light microscope.

### 2.6. Antibiotic Susceptibility Test

To examine antibiotic resistance of the bacterium, the strain isolated from the brain of diseased rainbow trout was used in the antibiotic susceptibility test. The strain was cultured on Mueller–Hinton agar and examined using the disc diffusion method [18]. Twenty eight antibiotics were used in the analysis and the results were analyzed as described by the Clinical and Laboratory Standards Institute [19]. The antibiotics used in the study were ampicillin, piperacillin, cefazolin, cefepime, cefotaxime, cefoxitin, ceftazidime, ceftizoxime, aztreonam, imipenem, meropenem, gentamicin, amikacin, kanamycin, streptomycin, tetracycline, doxycycline, ciprofloxacin, nalidixic acid, norfloxacin, ofloxacin, trimethoprim sulfamethoxazole, chloramphenicol, erythromycin, tobramycin, rifampicin, oxytetracycline, and ampicillin-sulbactam.

### 2.7. Challenge Trials

To determine the clinical symptoms, challenge trials were performed using S. xylosus strain SNU1 isolated from this study. Rainbow trout with an average weight of 10 g (± 3 g) were purchased from other trout farms located in Kangwon Province, Korea. The bacteria were grown in TSB for 24 h in a 25 °C incubator. The culture medium was centrifuged at 8000× *g* and washed with PBS. The titer of bacteria was calculated using an ocular density spectrophotometer (SmartSpec™ 3000; Bio-Rad, Hercules, CA, USA). The fish were intraperitoneally injected with the bacterial suspension at titer values of 6 × 10^7^, 10^6^ and 10^5^ CFU per fish, and the inoculum was suspended in 0.1 mL of PBS. Each group consisted of 10 fish and the trial was performed in triplicate. The same procedure was used for the control group, which was injected with PBS only. Each group of fish was maintained in 120-L water tanks individually and the temperature of the water was controlled at 15 °C using a titanium heater. The fish were observed for 15 days, and fish showing similar clinical symptoms of infection in the eyes were euthanized and selected to re-isolate the bacterium. Re-isolation of the bacterium was performed following the same procedure used for bacterial isolation: the fish organs, including the spleen, liver, kidney, and brain, were collected for examination. 

## 3. Results and Discussion

### 3.1. Examination and Diagnosis

Diseased fish collected for examination had clear fins and gills without any particular lesions such as hemorrhage, anemic appearance, and fin damage. Furthermore, the parasitic examination was negative. There were no signs of *Flavobacterium* infection on the fins and gills, when visually inspected. The PCR results for viral infections of IHN, IPN, and VHS were negative. As the mortality rate was low but continuous, bacterial infections were anticipated as the cause of death rather than viral infections. Whitish colonies of diameter 2–3 mm appeared on both TSA and BHI agar plates supplemented with homogenized samples of the brain, liver, and kidney. The plates supplemented with brain tissue homogenate showed a single type of colony and the other organ homogenates showed two to three types of colonies, which included a whitish colony as the dominant type. The whitish colonies were pure cultured for further identification of the bacterium on the TSA plates.

### 3.2. Identification of Bacteria and Phylogenetic Analysis

**Figure 2:**
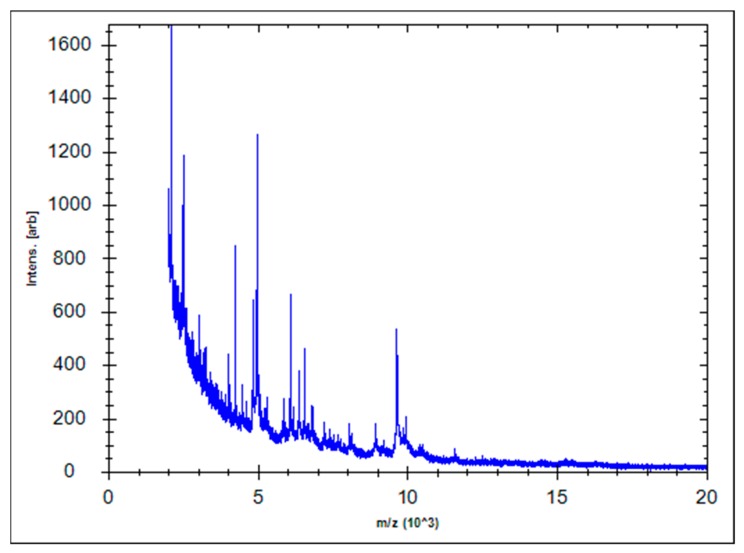
Spectra of *S. xylosus* generated using MALDI-TOF Bruker Biotyper. The y-axis represents the absolute intensity of the ions and x-axis represents the mass (m/z). The m/z value indicates the mass to charge ratio.

The complete 16S rRNA gene sequence of the bacterial strain has been deposited in the Genbank (accession number: MN294563), and from blast search and identification result of EzBioCloud server, we found that the strain was 99.86%, 99.80% and 99.80% similar to *S. saprophyticus* subsp. *saprophyticus* ATCC 15305T, *S. xylosus* CCM 2738T, and *S. edaphicus* P5085T, respectively. As the results of the 16S rRNA gene sequence analysis were similar, we performed additional identification using MALDI Biotyper 3(Bruker, Billerica, MA, USA). The spectral processing results showed that the signature peaks matched those of strain *S. xylosus* DSM 20267 with a score of 2022 (Figure 2). 

**Figure 3:**
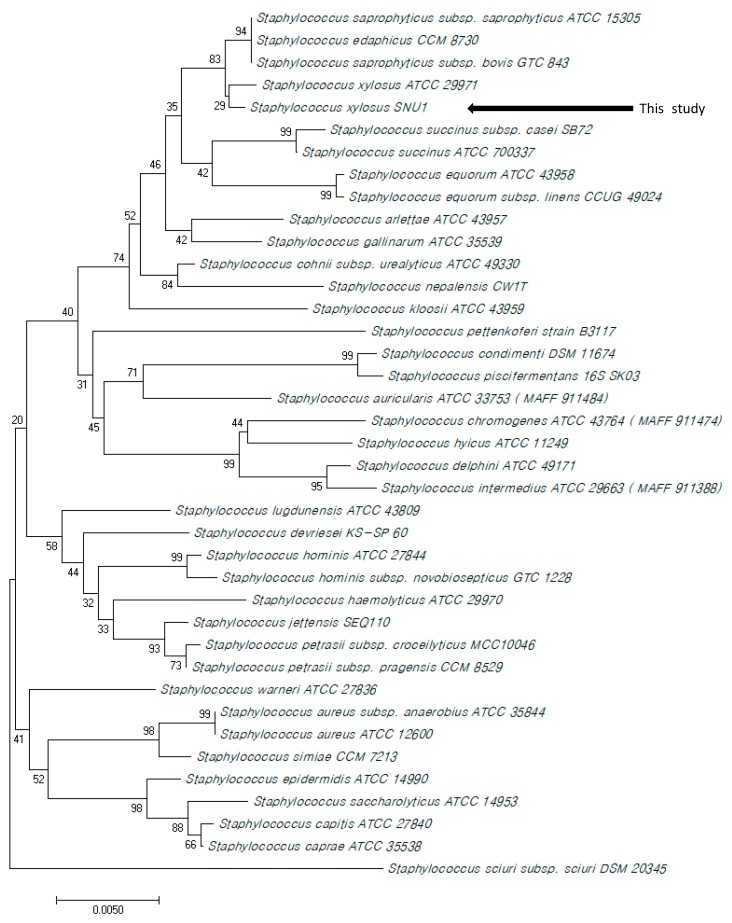
Phylogenetic tree constructed using partial 16S rRNA gene (1458 bp) of *Staphylococcus* spp. using the neighbor-joining method, bootstrap 1000, of MEGA 10.0 (GenBank accession number: MN294563).

To confirm the identification result, PCR was performed using specific primers designed to diagnose *S. xylosus* and *S. saprophyticus*. The PCR results showed that the bacterial strain was only positive for the *S. xylosus*-specific primer, which binds to its house-keeping gene (*sodA*). The phylogenetic tree constructed using 38 partial 16S rRNA genes of *Staphylococcus* spp. also showed that the bacterial strain belongs to the clade of *S. xylosus*, which supports the fact that the bacterial strain SNU1 is *S. xylosus* (Figure 3).

### 3.3. Histopathological Analysis

**Figure 4:**
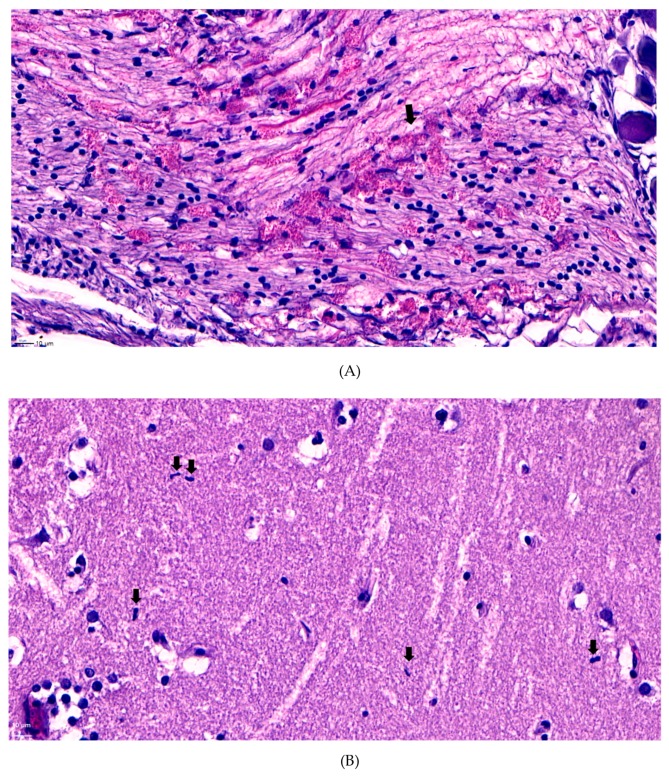

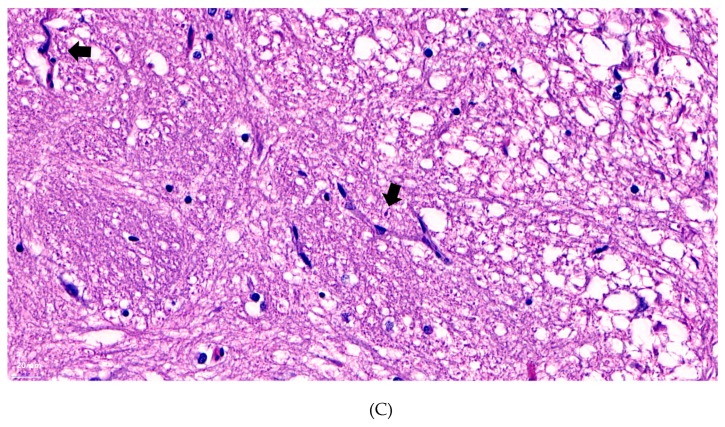
(**A**) Infiltration of eosinophilic granular leukocytes that are abundant along the cranial nerve root and ganglia (arrows defining eosinophilic leukcoytes lesion, bar: 10 µm). (**B**) Microglia observed in the optic lobe of diseased fish (arrows defining microglia, bar: 10 µm). (**C**) Signs of microgliosis observed in the optic lobe of the diseased fish (arrows defining signs of microgliosis, bar: 20 µm).

To determine the specific histological changes caused by *S. xylosus* infection in the optic nerves and brain, the brain was collected. Although, bacteria were also isolated from other organs, we only used the brain tissue for analysis, because we focused on the cause of eye proptosis symptoms, and not the other internal organs. We identified common histological changes in the cranial nerve roots and ganglia located near the optic lobe of diseased fish. The cranial ganglion and nerve roots clearly showed abundant eosinophilic granular leukocytes (Figure 4A) compared to those in the control group [20]. Moreover, we found microglia presented in the optic lobes (Figure 4B) and signs of gliosis (Figure 4C), which might have been caused by bacterial infection [21].

### 3.4. Antibiotic Susceptibility Test

To prevent and treat the disease, an antibiotic susceptibility test was performed using the disc diffusion method. As several *Staphylococcus* spp. are already known to be resistant to multiple antibiotics such as fluoroquinolones and linezolid, the bacteria strain was expected to be resistant to diverse antibiotics [3]. It was resistant to fluoroquinolones such as ciprofloxacin and ofloxacin and other antibiotics, but it was highly susceptible to tetracycline, oxytetracycline, doxycycline, and ampicillin-sulbactam (Table 1).

### 3.5. Pathogenicity Challenge Trial

To prove that the clinical symptoms were caused by a bacterial infection under Koch’s postulate, the challenged fish were maintained in 120 L water tanks and the water circulation was stopped to block the flow of pathogenic bacteria or other microorganism that can affect the results. With bacterial titers of 6 × 10^7^, 10^6^, and 10^5^ CFU per fish, the proportion of fish showing exophthalmia or eye protrusion was 66.67%, 33.33%, and 10%, respectively. The mortality rate was 23.33% at 6 × 10^7^ CFU per fish, and none at the other bacterial titers. This suggests that the bacterial infection can act as a primary pathogen interfering with the immune systems to cause a secondary infection. The mortality rate caused by *Staphylococcus* alone was very low, but as this infection can cause exophthalmia and induce other bacteria to flow by breaking the barrier of the immune system, the infection could be the primary cause of mortality. The control group did not show any signs of corneal infection and mortality was not observed during the trial period. Re-isolation of the bacterium was performed from moribund and euthanized fish with severe clinical symptoms. *Staphylococcus xylosus* strain SNU1 was re-isolated from the kidney, liver, and brain of the fish.

*Staphylococcus* spp. threaten human health globally, mainly because of their broad-spectrum antibiotic-resistance and zoonotic infection-causing ability [22]. Diverse staphylococcal infections have been reported in primates, domestic animals, livestock, and poultry in several countries [22]. They are normally present on the skin, nose, and mucous membranes of both healthy humans and animal, as well as diseased patients. Their pathogenic role depends on multiple infectious diseases of diverse severity. Due to their ability to gain an antibiotic-resistance gene easily, they cause numerous problems as a secondary infection pathogen in hospitalized patients and immune-suppressed animals. Moreover, the transmission of the species has gained great interest as it causes zoonotic infections, especially via food. 

Animal- and food-derived staphylococcal infections are of considerable importance and numerous studies have focused on food-transmitted bacterial infections and their effect on human health, such as meat- and fish-derived *S. aureus* infections. Therefore, bacterial infection of livestock and fish used for dietary purposes should be studied with respect to disease mechanism. There are some studies focused on staphylococcal infections in livestock and wild animals, but research in aquatic animals is scarce. Furthermore, there is a need to study antibiotic-resistance mechanisms when the bacterium first infects an animal and before being transmitted to humans. 

In the present study, we focused on *S. xylosus’s* infection mechanism and its pathogenicity in fish. To the best of our knowledge, this is the first study to report *S. xylosus* as a pathogen causing mortality and a primary pathogen to break the immune system barrier in rainbow trout. *Staphylococcus xylosus* infections have been reported to cause meningitis and keratitis in laboratory mice. Furthermore, its role as an opportunistic pathogen has been confirmed in various animals. Based on these findings, in the present study, we verified the pathogenicity and infection mechanism of *S. xylosus* in rainbow trout, and investigated a method for preventing associated diseases using antibiotics. The most appropriate antibiotic for use in aquaculture was considered as oxytetracycline which is one of the most used antibiotic in fisheries. Furthermore, the bacterium was cultured at 37 °C to verify whether it can act as a zoonotic pathogen, and the results confirmed that it can grow at temperatures of up to 40 °C. The pathogenicity of the bacteria was not performed for mammals in our study, but since rainbow trout were mostly consumed as raw fish in Korea, the consumer may avoid the risk of bacterial transmission by judging clinical symptoms of rainbow trout described in our study such as keratitis, eyeball turbidity, and exophthalmia. Therefore, further investigation regarding the transmission of the bacterium via fish and its effect on human health is needed. This is due to the fact that in several Asian countries including Korea, rainbow trout is consumed raw, which can cause serious problems in immune-suppressed individuals, if the bacterium is a zoonotic pathogen. Since we only performed growth experiments at a certain temperature in this study, there are limitations to ascertain the pathogenicity of this bacterium in human. However, since our study indicates a pathogenic role for *S. xylosus* in rainbow trout, there should caution when considering the zoonotic role of the bacterium. 

## 4. Conclusions 

To conclude, our study focused on the S. xylosus infection in rainbow trout fisheries of Korea which induced mortality and broke primary immune barrier of the fish due to exophthalmia. This is first case reporting S. xylosus infection in fish and possibility of the bacterium to act as a pathogen in aquaculture. Since antibiotic resistance test indicated appropriate antibiotics for the usage in fisheries, this may not be a serious problem in the present period, but as *Staphylococcus*, family were known to having multi antibiotic resistance ability, further study about its resistance mechanism seems to be needed. In addition, because rainbow trout were mostly consumed as raw fish in many Asian countries, its risk for the transmission from food to human needs to be acknowledged for public health. 

## Figures and Tables

**Table 1:** Results of the antibiotic susceptibility test using the disk diffusion method.

Antibiotic	Resistance	Inhibition Zone Diameter (mm)	Antibiotic	Resistance	Inhibition Zone Diameter (mm)
Ampicillin	I	15	Piperacillin	R	15
Cefazolin	R	16	Cefepime	R	5
Cefotaxime	R	11	Cefoxitin	R	14
Ceftazidime	R	13	Ceftizoxime	R	3
Aztreonam	R	2	Imipenem	I	19
Meropenem	R	17	Gentamicin	R	6
Amikacin	R	8	Kanamycin	R	13
Streptomycin	R	11	Tetracycline	S	25
Doxycycline	S	25	Ciprofloxacin	R	10
Ciprofloxacin	R	1	Norfloxacin	R	9
Ofloxacin	R	9	Trimethoprim Sulfamethoxazole	I	16
Chloramphenicol	I	15	Erythromycin	I	16
Tobramycin	S	28	Rifampicin	R	10
Oxytetracycline	S	25	Ampicillin Sulbactam	S	24

R: resistant, I: intermediate, and S: susceptible.
